# Learning and memory under stress: implications for the classroom

**DOI:** 10.1038/npjscilearn.2016.11

**Published:** 2016-06-29

**Authors:** Susanne Vogel, Lars Schwabe

**Affiliations:** 1 Department of Cognitive Psychology, Institute of Psychology, University of Hamburg, Hamburg, Germany

## Abstract

Exams, tight deadlines and interpersonal conflicts are just a few examples of the many events that may result in high levels of stress in both students and teachers. Research over the past two decades identified stress and the hormones and neurotransmitters released during and after a stressful event as major modulators of human learning and memory processes, with critical implications for educational contexts. While stress around the time of learning is thought to enhance memory formation, thus leading to robust memories, stress markedly impairs memory retrieval, bearing, for instance, the risk of underachieving at exams. Recent evidence further indicates that stress may hamper the updating of memories in the light of new information and induce a shift from a flexible, ‘cognitive’ form of learning towards rather rigid, ‘habit’-like behaviour. Together, these stress-induced changes may explain some of the difficulties of learning and remembering under stress in the classroom. Taking these insights from psychology and neuroscience into account could bear the potential to facilitate processes of education for both students and teachers.

Stressful events are very common in educational settings, both for students and for teachers. A multitude of exams, evaluations and deadlines creates an enormous pressure to perform. This stress, however, can have a critical impact on learning and memory processes,^1,2^ which are at the heart of our educational system. Beyond their relevance in educational contexts, stress-induced alterations in learning and memory are also thought to contribute to stress-related mental disorders, such as major depressive disorder or post-traumatic stress disorder.^3^ Therefore, a large number of studies has been conducted to better understand how stress affects learning and memory. The effects of stress were found to be complex, though, with stress having both enhancing and impairing effects on memory, depending on the specific memory process or stage that is affected by stress^1,4^ and the activity profile of major physiological stress response systems.

This review summarises the current state of knowledge on the impact of (acute) stress on memory and derives implications for educational settings from these laboratory findings. Because our focus is on memory processes most relevant in the classroom, we will concentrate mainly on the effects of (moderate) stress (induced in laboratory settings) on episodic and semantic memory, as well as the engagement of multiple memory systems in healthy humans (for reviews on the influence of stress on other forms of memory or other cognitive processes, see e.g. Arnsten^5^ and Sandi^6^). As the influence of stress on learning and memory is intimately linked to the physiological and endocrine changes initiated on a stressful encounter, we will cover these changes first. Next, we will provide a concise overview of how stress, through the action of major stress mediators, induces time-dependent changes in how much information is learned, consolidated and retrieved (i.e., memory quantity). In the third part of this review, we will discuss recent findings on how stress may change the dynamics of memories, their updating in the face of novel information, and the integration of new knowledge into existing memories, all key processes in educational settings. We will then highlight the impact of stress on the engagement of different memory systems, arguing that stress effects are not limited to how much we learn or remember but that stress also changes the nature (or quality) of memories, for instance, the strategies that are used during learning. Based on these empirical findings, we will finally discuss the implications of stress effects on learning and memory processes for the classroom.

## The well-coordinated physiological response to stressors

**Figure 1:**
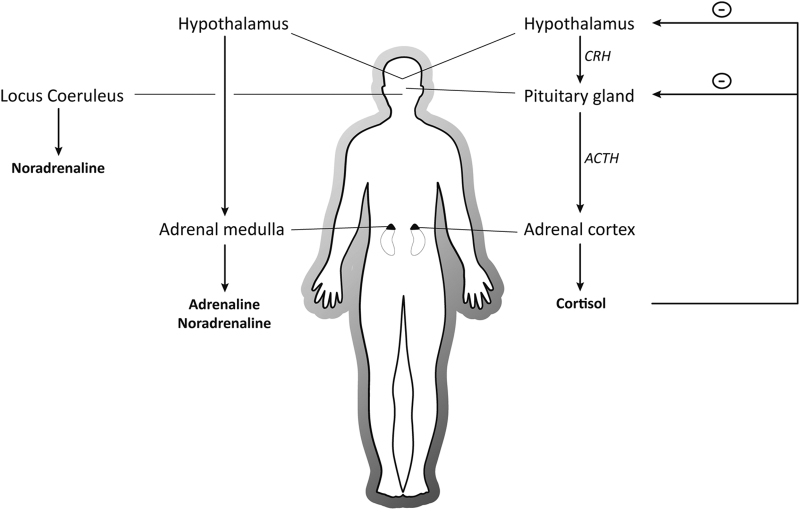
Systems activated in response to stressful events. On a stressful encounter, the autonomic nervous system (left) is activated within seconds to release catecholamines (e.g., noradrenaline) from the adrenal medulla and the locus coeruleus in the brain stem. Catecholamines are implicated in the ‘fight-or-flight’ response, but they also have profound effects on attention, working memory and long-term memory. Somewhat slower, the hypothalamus–pituitary–adrenal axis is activated, releasing corticotropin-releasing hormone (CRH) from the hypothalamus which stimulates the anterior pituitary to secrete adrenocorticotropic hormone (ACTH). ACTH in turn causes the adrenal cortex to produce cortisol and release it into the blood stream. Cortisol reaches peak level concentrations ~20–30 min after stress onset and readily enters the brain to affect cognition and behaviour. Cortisol feedback to the pituitary, hypothalamus and other brain areas (e.g., the hippocampus) prevents the system from overshooting.

Difficult situations in the classroom such as exams or interpersonal conflicts can challenge or exceed the coping strategies or resources available and thus threaten our homoeostasis, our inner balance, leading us to feel ‘stressed’.^7^ The individual appraisal of the situation is critical as it determines the response that follows.^8,9^ If a situation is appraised as stressful, a well-described cascade of physiological and endocrine changes is set in motion in order to re-establish homoeostasis and to promote long-term well-being.^10^ Although this stress response is very complex with numerous mediators involved, two major stress systems appear to be critical for the modulation of learning and memory processes, the rapid autonomic nervous system (ANS) and the slower hypothalamus–pituitary–adrenal axis (Figure 1). Within seconds, the ANS is activated, leading to the release of catecholamines such as noradrenaline (NA), both from the adrenal medulla and the locus coeruleus in the brain.^10^ Catecholamines prepare the body for ‘fight-or-flight’ responses and rapidly affect neural functioning in several brain regions critical for learning and memory, such as the hippocampus, amygdala and prefrontal cortex (PFC).^5,11^ Somewhat slower, a second system is activated in response to stress, the hypothalamus–pituitary–adrenal axis, resulting in the release of corticosteroids (in humans mainly cortisol) from the adrenal cortex. Cortisol reaches peak level concentrations ~20–30 min after stressor onset,^10^ readily enters the brain and binds to two different receptors to induce its effects on cognition: The glucocorticoid receptor (GR) is expressed ubiquitously throughout the brain, whereas the mineralocorticoid receptor (MR) is mainly expressed in brain regions related to memory and emotion, for instance, the hippocampus, amygdala and PFC.^12,13^ On binding to these receptors, cortisol operates via two different modes of action, a non-genomic, often MR-mediated mode develops rapidly^14^ and enhances neural excitability in the amygdala and hippocampus,^15,16^ presumably supporting memory formation. This rapid mode is followed by a slower, often GR-dependent mode that is assumed to develop ~60–90 min after stressor onset and to involve longer-lasting changes to DNA translation and transcription.^17^ The slow genomic mode is assumed to revert the acute effects of stress and to re-establish homoeostasis by decreasing neural excitability in the amygdala and hippocampus long after stress.^4^

**Figure 2:**
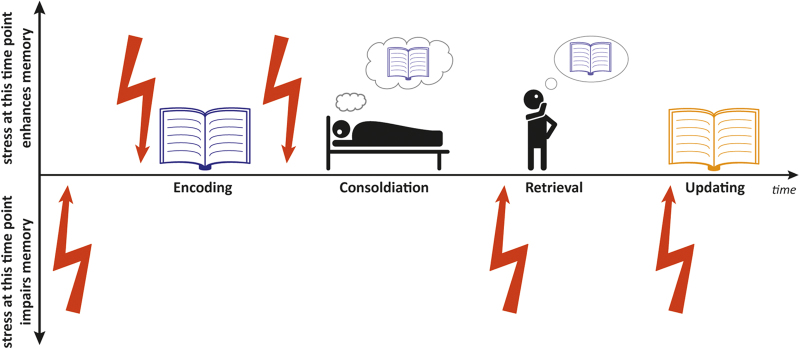
The effects of stress on memory depend on the specific memory process investigated and the temporal proximity between the stressful event and this memory process. While stress (indicated as red flash) long before encoding impairs memory formation, stress shortly before or after the presentation of new information generally enhances subsequent memory performance. In sharp contrast, stress before memory retrieval impairs the recall of information learned previously which may directly affect performance at exams. In education, knowledge needs to be frequently updated by new facts or concepts relating to prior knowledge. In addition to its effects on memory encoding and retrieval, stress appears to impair this integration of new information into existing knowledge structures.

This striking temporal profile of the stress response leads to differential effects of stress on learning and memory, depending on the temporal proximity between the stressful event and the memory process investigated. For instance, stress experienced just before memory retrieval, when catecholamine levels are still high and cortisol levels are not elevated yet, may have very different effects than stress experienced 90 min before retrieval, when catecholamine levels returned to baseline and genomic cortisol actions are at work.^18,19^ Moreover, distinct memory stages, i.e., encoding, consolidation or retrieval may be differently affected by these time-dependent physiological changes after a stressful encounter. In the next section, we will portray the time-dependent effects of stress on learning and memory, taking into account both the specific memory stage affected and the temporal proximity between the stressful event and the memory formation or retrieval process (Figure 2).

## Time-dependent effects of stress on memory quantity

Emotionally arousing events are typically very well-remembered. Likewise, individuals who experienced extremely stressful (traumatic) events may suffer from very vivid memories of these events, suggesting that severe stress during or just before encoding may boost memory formation. In line with these observations, studies showed that also lower levels of stress (as they may occur more frequently in schools) during or just before learning may strengthen human memory.^20–23^ This effect of stress on encoding was often stronger for emotional compared with neutral learning material.^24^ Another factor moderating the influence of stress on learning is the correspondence between the stressful context and the learning material. For example, stress during learning specifically enhanced memory for material that was related to the context of the stressful task and thus putatively more relevant.^20^ Material that is unrelated to an ongoing stressor, however, is typically not very well-remembered later on.^25^ Despite many studies showing a stress-induced learning enhancement if stressor and learning coincide, some studies found the opposite effect.^26,27^ This divergence might be due to other factors than just the timing of the stressful encounter, such as differences in the interval between study and retrieval or individual differences due to sex, genetics or the developmental background.^28–31^ In sum, being moderately stressed can enhance memory formation for emotional material and information that is related to the stressful context, whereas stress may impair the encoding of stressor-unrelated material.

At the neural level, catecholamines such as NA appear to play a critical role in the enhancing effects of stress or emotional arousal on learning. Studies in rodents demonstrated that NA exposure strengthened synaptic contacts in the hippocampus^11^ and that the concentration of NA in the amygdala after encoding predicted memory strength.^32^ Corticosteroids, however, appear to play an important role as well. For instance, MR-activation rapidly enhanced neural excitability in the amygdala and hippocampus which may further aid successful memory encoding.^15,16^ Additional evidence for a role of corticosteroids came from human pharmacological studies, demonstrating that the administration of 20 mg cortisol prior to learning boosted later memory, especially for emotionally arousing pictures.^33^ Notably, this memory advantage for emotional material depends on NA, as it can be blocked by the beta-blocker propranolol.^34^ Human neuroimaging studies then set out to elucidate the neural mechanism underlying the stress-induced learning enhancement. The immediate release of NA under stress activated a network of brain regions known as the salience network encompassing the amygdala, anterior cingulate cortex and anterior insula.^35,36^ This rapid upregulation of the salience network allowed enhanced vigilance and better processing of threat-related information which may improve memory encoding in stressful situations. Some minutes later, the release of cortisol reduced global signal in the electroencephalogram (EEG), which was interpreted as a reduction in background processing in order to allow efficient processing of relevant information by enhancing the signal-to-noise ratio.^37^ In line with an enhanced processing of important information, the stress-induced increase in processing and encoding of study items in the brain was related to better memory performance for these items at test.^38,39^ Several studies also investigated the interplay of NA and cortisol in memory encoding. Supporting evidence for such an interaction came, for instance, from a study showing that emotional learning material activated the amygdala, an effect that depended on NA availability as it was abolished by propranolol.^40^ Importantly, this amygdala response to emotional stimuli was particularly prominent in those individuals with higher cortisol levels during encoding.^41^ Moreover, the combined administration of cortisol and yohimbine, a drug increasing NA stimulation, switched neural activity towards a strong deactivation of prefrontal areas,^42^ potentially releasing the amygdala from inhibitory top-down control and improving memory encoding.

While stress around the time of learning enhances memory, stress (or cortisol administration of 25 mg) long before learning or in a distinctly different context does not promote new learning^43^ and can even hinder successful encoding of new information.^21^ For example, while stress directly before learning enhanced later recognition memory, memory was impaired if stress was experienced 30 min before learning.^21^ This memory impairing effect of stress long before learning has been associated with a decrease in neural excitability in the hippocampus long after cortisol administration,^44^ which might suggest that genomic actions of cortisol protect the consolidation of information learned during the stressful encounter.^2^ In line with this finding of decreased hippocampal excitability, cortisol administered more than 1 h before MRI measurements reduced hippocampal and amygdala activity in humans,^45,46^ possibly impairing the formation of new memories. In the same time period, the activity of the salience network decreased again to pre-stress levels while activity in the executive control network increased,^35^ allowing the individual to recover from the stressful situation and to re-approach homoeostasis. However, there is evidence that this reversal of heightened salience network activity, which is important for higher cognitive control functions to improve coping in the aftermath of stress, does not occur when the participants remain in the stressful context. For instance, the coupling between the amygdala and the salience network remained enhanced after 1 h if the participants were still in the context of the stress induction procedure,^47^ again highlighting the role of context as a moderator of stress effects on learning.

When stress is experienced before or during a learning episode, its effects on memory encoding can hardly be dissociated from those on memory consolidation. Also in educational settings, influences of stress on memory encoding can often not be separated from those on memory storage. However, by administering stress or stress mediators shortly after learning, thus excluding an influence on memory encoding, experimental studies were able to isolate stress effects on memory consolidation. Several studies in humans showed that stress or adrenaline injections shortly after learning improved memory consolidation, an effect which was more pronounced for emotionally arousing material,^26,48, 49, 50^ highlighting the importance of the emotionality of the study material. Studies in rodents also demonstrated that the administration of NA or corticosteroids just after learning improved consolidation,^51^ and that this enhancing effect (at least on hippocampal memory) required the interaction between NA and GR-mediated cortisol effects in the amygdala.^52–55^

**Figure 3:**
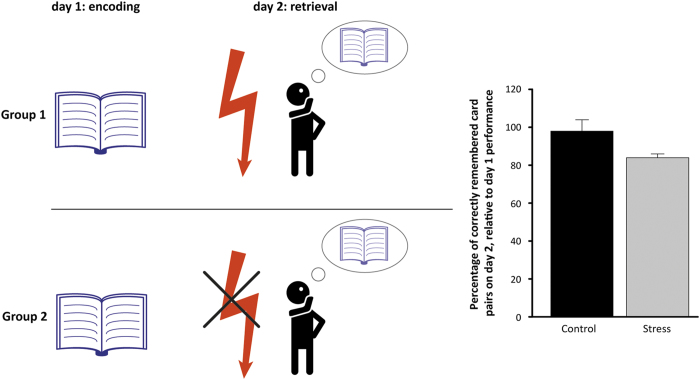
Stress impairs memory retrieval. Participants learned a two-dimensional object location task similar to the game ‘concentration’ (note that for illustrative purposes encoding is depicted by a book, similar to studying in class). One day later, participants either underwent a mild stress induction procedure (indicated by the red flash) or a non-stressful control procedure before recalling the card pair locations learned on day 1. Participants in the stress group recalled significantly fewer card pair locations on day 2 than participants in the control group (relative to their performance on day 1), indicating that stress before retention testing reduced memory performance. Adjusted, with permission, from ref. 63.

The effects of stress on memory are, however, not limited to the formation of memories (i.e., memory encoding and consolidation) but extend also to memory retrieval. Given that exams and tests can easily cause stress in students and students are evaluated based on their performance in these tests, it is particularly relevant to understand how stress affects memory recall. In line with seminal findings in rodents,^56^ many studies in humans demonstrated that acute stress impaired memory retrieval after a stressful encounter (refs 18,19,57,58,59 but see refs 60,^61^). Retrieval in the stressful situation itself seemed not to be affected or even enhanced,^18,19^ particularly when retrieval performance was directly relevant to the stressful encounter. Retrieval more than 20 min after stress, however, when cortisol levels were already elevated, was impaired by the cortisol response to stress^18,19,58^ (Figure 3) and the impairment appeared to be even stronger at a time point when genomic cortisol actions had developed,^18^ suggesting that the impairing effects of stress can last much longer than previously known. This retrieval deficit after stress was not only found in adults but was also observed in 8–10-year-old children, highlighting the relevance of these findings for educational settings.^59^ The disrupting effect of stress on retrieval was stronger for emotional material^26,62^ and also the context appeared to play a moderating role on the effects of stress on retrieval. For instance, if the retrieval test was relevant for the stressful situation or if both learning and test took place in the same context, so that the context served as a retrieval cue, recall was spared from the impairing effects of stress.^19,63^

The negative effect of stress on retrieval could be mimicked by administering a GR agonist and blocked by the cortisol synthesis inhibitor metyrapone in rodents, which suggests a GR-dependent pathway^43,56,64,65^ reducing blood flow in the medial temporal lobe.^66^ However, the interaction with NA appears to be crucial as the impairing effects of cortisol depended on noradrenergic activation of the amygdala.^52^ For instance, blocking the action of NA pharmacologically with propranolol abolished the impairing effect of cortisol on emotional memory retrieval.^67^ Thus, similar to memory consolidation, the interaction between GR-mediated cortisol action and NA appears to be crucial for stress-induced effects on memory retrieval.^67^

To summarise, stress affects memory in a time-dependent manner, often enhancing memory formation around the time of the stressful encounter but impairing memory retrieval and the acquisition of information encoded long after the stressful event. These effects depend on interactions between NA and cortisol in the amygdala and are thus often stronger for emotional than for neutral learning material. In the next paragraph, we will move beyond stress-induced changes in memory performance and describe how stress may also affect the integration of new information into existing memories, i.e., knowledge updating.

## Stress and the dynamics of memory

Very often, students are not only required to recall study material, but to integrate new information into existing knowledge structures. In fact, integrating new information into existing memories is a key process in education (as well as in life in general where we are constantly required to update our knowledge). Such updating implicates that memories remain malleable even long time after they have been formed initially and research over the past 15 years shows that this is indeed the case (for review, see ref. 68). There is compelling evidence that consolidated, seemingly stable memories return to a labile state when they are reactivated,^68–71^ which requires the re-stabilization of those memories in a process called reconsolidation. During reconsolidation, the reactivated memory can be weakened, strengthened or altered.^69,71^ In other words, reconsolidation most likely represents the mechanism underlying memory updating processes.^72^ As reconsolidation involves the hippocampus^71^ and the PFC,^73^ areas that are main targets of stress modulators, it seems reasonable to assume that stress would also affect reconsolidation. First evidence for such stress effects on reconsolidation came from rodent studies showing that stress or cortisol injections after memory reactivation impaired subsequent memory expression, suggesting that stress impaired reconsolidation.^74,75^ For instance, stress after reactivation of a memory trace interfered with performance at a later memory test, an effect which depended on GR-mediated cortisol activity in the amygdala.^75^ Several studies in humans support the hypothesis that stress can affect memory reconsolidation and thus memory updating, yet the specific conditions leading to impairing or enhancing effects of stress on reconsolidation are still under investigation.^76–78^

**Figure 4:**
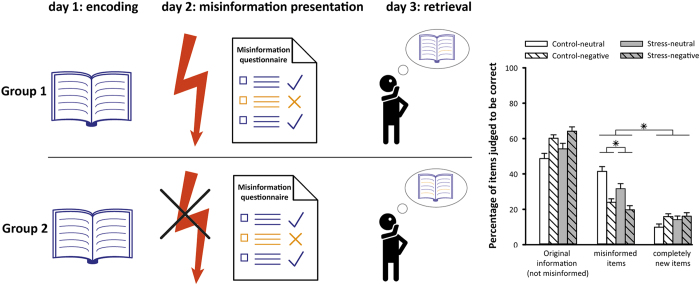
Stress reduces the integration of new information into existing memories. On day 1, participants were instructed to memorise different stories presented in short movie clips (note that encoding is illustrated by a book for illustrative purposes). On day two, participants either underwent a mild stress induction procedure (indicated by the red flash) or a non-stressful control procedure before they were presented with a questionnaire regarding the study material from day 1. Importantly, some items of this questionnaire included wrong information about the study material (misinformation, shown in orange). On day 3, forced choice questions were used to test whether the misinformation had been integrated into the memory trace of the study material. In the memory test, possible answers were the correct original information, the misinformation presented the day before and other incorrect answers (lures) that were not referred to on day 2. Overall, participants endorsed misinformation more often than lures, thus demonstrating a misinformation effect. Critically, stressed participants endorsed fewer misinformation items than participants of the control group, suggesting that stress reduced the modification of the original memory on day 2. Adjusted, with permission, from ref. 81. *P<0.005

Further evidence for a critical role of stress in the updating of memories comes from studies on the so-called misinformation effect. This effect describes the incorporation of misleading information presented after encoding the original event into the memory for this event.^79^ Although this effect mainly concerns the biasing influence of misinformation on memory, it provides important insights into memory updating in general and studying how stress affects the misinformation effect may allow a deeper understanding of how stress affects the updating of memories. For instance, it was shown that if highly arousing information is learned during stress, this resulted in more robust memories that were less vulnerable to being ‘updated’ by subsequent (mis)information.^80^ Similarly, misinformation was less often incorporated into existing memories if the participants were stressed before the presentation of misinformation, thus indicating that stress interferes with the updating of the existing memory trace^81^ (Figure 4). As the mechanism underlying the misinformation effect is assumed to be reconsolidation,^72^ this finding is in line with reports showing an impairing effect of stress on memory reconsolidation.^74,75,78^ In sum, there is accumulating evidence that stress may interfere with the updating of memories, which may have negative implications for education where new information often has to be incorporated into existing knowledge.

## Stress alters the way we learn: effects on memory quality

**Figure 5:**
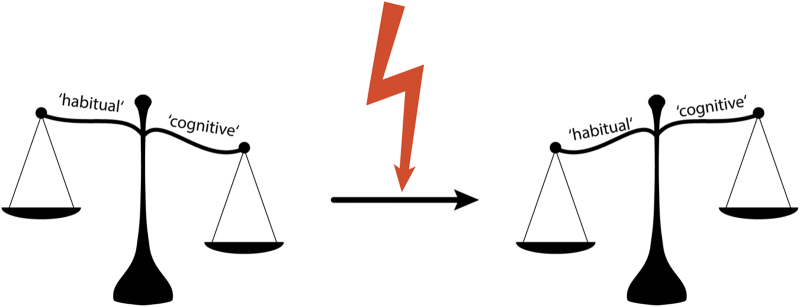
Stress shifts the balance between multiple systems underlying learning and memory. At rest, this balance is tilted towards the ‘cognitive’ memory system depending on the hippocampus, allowing for the formation and recall of flexible memories. Stress, however, is thought to alter the system domination learning and memory. Under stress (indicated by a red flash), the balance tips towards more rigid ‘habit’ memories encoded by the dorsal striatum. Thus, stress affects not only how much is learned (memory quantity) but also what is encoded and how memories are built (memory quality).

Most studies investigating the effects of stress on memory encoding, retrieval or updating focused on memories encoded by the hippocampus. However, experiences can be encoded by multiple memory systems operating in parallel, differing in their neural substrate and in the information processed.^82,83^ Several studies demonstrated that stress has a critical impact on which of these memory systems is used to form and retrieve memories, implicating that stress changes the nature or quality of memories^84,85^ (see Figure 5). Early studies in rodents demonstrated that stress or amygdala activation through anxiogenic drugs at encoding induced a shift from a flexible ‘cognitive’ memory system depending on the hippocampus towards a more rigid, ‘habit’-like memory system based on the dorsal striatum.^82,86,87^ Thus, under stress, more rigid stimulus–response associations are learned rather than complex representations of our environment including the relationship between stimuli or task requirements. This shift in the system that controls memory could be blocked by an MR-antagonist, suggesting that the shift is due to MR-mediated cortisol action.^88,89^ Importantly, stress itself did not disrupt learning, but blocking the shift towards habit memories markedly impaired performance,^88^ suggesting that the shift towards the striatum-based habit system is adaptive and beneficial for performance under stress. So far, only one study investigated whether this stress-induced shift also affects memory retrieval, and indeed anxiogenic drugs injected into the amygdala before retrieval biased rats towards an increased use of their dorsal striatum at the expense of the hippocampal memory system.^90^ To summarise, these studies in rodents suggest that stress induces a qualitative shift in the systems guiding learning (and, most likely, retrieval), from a cognitive, hippocampus-dependent memory system towards a habit-like memory system based on the striatum.

In line with these rodent findings, stress shifts the systems dominating memory encoding also in humans towards an increased use of striatal habit-like memory, at the expense of hippocampal memory.^91–93^ For example, stressed participants often used a habit-like striatal learning strategy instead of a hippocampal strategy to solve a learning task.^93^ Similar to the findings in rodents, stress did not affect learning performance *per se* if participants switched to the striatal memory system,^91^ yet performance was impaired when participants tried to recruit the hippocampal memory system despite stress.^93^ Accordingly, task performance was positively correlated with hippocampal activity in non-stressed control participants, whereas performance correlated positively with striatal activity and even negatively with hippocampal activity in stressed participants.^93^ The amygdala appeared to orchestrate this stress-induced shift by rapidly increasing functional connectivity with the dorsal striatum and decreasing its coupling with the hippocampus.^94,95^ Importantly, an MR-antagonist blocked the stress-induced shift both at the behavioural and neural level,^94,95^ demonstrating that the stress-induced shift appears to depend on cortisol acting via the MR.^89^

In addition to the shift from hippocampal to striatal memory, stress affects the balance between memory systems underlying instrumental behaviour, i.e., behaviour aimed at obtaining rewards or avoiding punishments. Learning and performing these actions can be controlled by a ‘habitual’ system relying on the dorsolateral striatum which acts largely independently of the current value of the action-outcome, or a ‘goal-directed’ system depending on the PFC, dorsomedial striatum, and dorsomedial thalamus which is sensitive to changes in outcome value.^96^ Under stress, human and rodent behaviour is rendered more habitual and based on stimulus–response associations rather than action-outcome associations which underlie goal-directed actions.^97–101^ Moreover, the behaviour of stressed individuals was more resistant against extinction procedures,^92^ further highlighting the rigid, rather habitual behaviour of stressed individuals. For example, stressed infants continued to use habit actions even though the behaviour was not reinforced anymore, whereas non-stressed infants stopped showing the behaviour when the reinforcement ended.^100^ The stress-induced modulation of instrumental behaviour can be abolished by beta blockers, suggesting that NA plays a crucial role in this shift towards habit behaviour.^98^ Again, NA appears to interact with the effects of cortisol as the stress-induced shift towards habits can be mimicked by the combined administration of cortisol and yohimbine,^97^ and beta-adrenergic blockade by propranolol prevents the stress-induced bias towards habits.^98^ In the brain, this shift has been associated with a reduced sensitivity of the orbitofrontal and medial PFC to changes in outcome value, whereas brain regions implicated in habit learning were not affected.^99^

To summarise, stress cannot only affect how much information we learn and remember, but stress also flips the balance between the systems dominating learning and memory, which has considerable consequences for the nature and flexibility of memories and the goal-directedness of behaviour.

## Stress and memory in the classroom

School children often encounter stressful events inside and outside of their school environment^102^ and nearly 70% of primary school children report symptoms of stress such as worries, anxiety or sadness.^103^ In the preceding chapters, we argued that situations appraised as stressful have strong and diverse effects on human memory. While learning during or immediately after stress is often enhanced, stress disrupts memory retrieval and updating, and these effects are most pronounced for emotionally arousing material. Finally, we argued that stress shifts the balance between multiple systems underlying memories and instrumental behaviours towards the formation and recall of rather rigid habit-like memories. Together, these findings highlight that stress may critically shape our memories, which is of utmost importance in all educational contexts.

In the classroom, these stress effects on memory may have far-reaching consequences for students. For instance, emotions or light to moderate forms of stress (i.e., cognitive challenges without excessive demands or moderate emotional arousal that results, e.g., from hearing something that is unexpected) may increase memory formation, which may have positive effects on memories for study material. Yet, these effects likely follow an inverted u-shape and can revert with too high levels of stress.^28,104^ Moreover, stress may lead to stronger memories for negative events happening in the classroom, such as failed exams, embarrassing experiences or interpersonal conflicts (e.g., bullying) and these strong negative memories may induce long-lasting frustration and a negative attitude towards school and the individual’s abilities. These negative consequences of stress on students may be intensified by the deleterious effects of stress on memory retrieval. Moderate or high levels of stress before exams will most likely hinder memory retrieval and lead to an underestimation of the students’ knowledge, putatively resulting in bad grades. Furthermore, stress may hinder the integration of new information into existing knowledge structures, which may prevent the updating of knowledge by new facts or a deep multidisciplinary understanding of concepts which is often required in education. Finally, by altering the balance between memory systems, stress may lead to strong, rigid memories and the retrieval of habits rather than creative and complex solutions to new problems, which may again lead to an underestimation of the students’ abilities.

Although the effects of stress on memory are highly relevant to students, also teachers frequently encounter stressful events and >40% report high levels of work stress.^105^ Also for teachers, appraising events as stressful may lead to strong negative memories of unpleasant situations in the classroom with implications for their work attitude and potentially their mental health. Moreover, stress may impair the quality of teaching if the teacher’s flexibility is decreased, which might hamper adaptive responding to the individual needs and resources of students. Instead, habitual procedures may be supported by stress, leading to a more repetitive teaching style, which may in turn lead to more problems in class.

Considering this wide range of possible stress effects in educational settings, strategies to deal with stress and its consequences are needed. First and foremost, teachers should be aware of the impact stress may have on memory formation, retrieval and updating. Moreover, students should be educated about the influence of stress on memory to raise awareness for the powerful effects stress may exert and the need for efficient coping strategies. It is important to note that potentially stressful events do not necessarily lead to a stress response, but that the individual appraisal of the situation and the available coping strategies determine whether a situation results in the activation of stress systems or not. This dependence on appraisal and coping can explain why some individuals suffer much less from potentially stressful circumstances than others. Thus, next to changing potentially stressful situations, students should be educated about effective coping strategies.^8,106^

Furthermore, based on findings demonstrating that emotional material is typically better remembered than neutral material, an emotional component (mainly positive) may be added while students learn new information to enhance later memory.^21,23,24,33,49,107,108^ For example, this could be achieved by explicit positive verbal reinforcement of students during learning in class. Furthermore, movie clips might be used which do not only focus on the learning material itself, but place it into an emotional context, e.g., by making the links to the student and his or her everyday life explicit.

To counteract the strong negative effects of stress on memory retrieval and updating, strong stressors before exams or before new information is presented to update students’ knowledge should be avoided as far as possible. To reduce stress, practice exams may familiarise the students with the exam situation and trainings in stress reduction techniques or other coping strategies might help students to alleviate stress symptoms. Teachers should also be aware that different forms of retrieval may be differentially affected by stress. Free recall seems to be disrupted more easily by stress than cued recall,^62^ suggesting that recall cues may enhance the chance that students can actually retrieve the information they have learned. It is important to note that the impairing effects of stress on retrieval are quite long-lasting, such that stressors long before the exam (e.g., at home) may still affect performance in the test situation. Therefore, children with trouble at home or frequent stressful life events may need special attention before exams to reduce the effects of stress.

Stress does not only induce a deficit in memory retrieval and memory updating, it also changes the way information is stored and retrieved by multiple memory systems. Stress before learning may bias students towards rigid forms of learning, which may hinder the successful transfer of knowledge and reduce cognitive flexibility in problem solving. However, the negative effects of stress on memory retrieval may be counteracted to some extent by thoroughly and repeatedly practicing useful routines which can be recalled rather automatically. This may be especially relevant for the training of correct actions during emergency situations. For instance, given that flexible memory recall and knowledge application is hindered under stress, pilots or physicians should be trained extensively in the correct routines they should apply in stressful emergency situations. If these procedures are automatised, it is much more likely that they can actually be retrieved and translated to behaviour.

Last, students and teachers should be aware of the powerful effects of context. It has been shown repeatedly that memory is enhanced when learning and recall take place in the same context as the context serves as a strong retrieval cue.^109^ Moreover, although stress often impairs retrieval, this effect seems to be alleviated if learning and retrieval context match, indicating that the effect of context might counteract stress-induced memory impairments.^63^

## Conclusion and outlook

Stress has far-reaching consequences on our ability to learn and remember, with major implications for educational settings. Considering that stress is ubiquitous in education and even primary school children often report stress symptoms, understanding the effects of stress on memory is very important. For one, an optimised education is of utmost importance for the individual, laying the foundation of later career success and socioeconomic status. In addition, our educational system is highly relevant for society as a whole by building and instructing the next generation.

Despite the striking advances the field has seen in our understanding of how stress changes learning and memory processes, several questions remain to be answered, e.g., concerning interindividual differences in the effects of stress on memory. While some studies suggested that differences in personality, gender or stress system reactivity may moderate how stress affects learning,^28^ the findings are not conclusive yet and the involved mechanisms are not understood sufficiently well to derive recommendations for teachers. Understanding these interindividual differences is a key to personalised approaches or training programmes directed at preventing stress-induced impairments. In addition, more research is necessary to understand the precise development of stress effects on memories over time as it is currently unclear when exactly the enhancing and impairing effects of stress on memory formation arise and how long they last. Likewise, it is currently not well-understood whether different types^110^ or intensities^104^ of stressors have different effects on memory. Furthermore, most studies did not explicitly distinguish between stress effects on different types of declarative memory, i.e., semantic and declarative memories. Future studies are required to assess whether stress has differential effects on these memory systems, which would provide important insights into how stress changes different forms of learning and memory. Finally, the exposure to prolonged or repeated stress, as well as stress during critical periods of brain development may also have strong effects on learning and memory in children which need to be better understood to counteract the impairments they may cause.^111^ Thus, different intensities of stress at different time points during development may induce different effects which remain to be further investigated. Future research on the effects of stress on learning and memory will hopefully answer these and related questions and thus further deepen our understanding of how stress affects memory and why individuals differ in response to stress. Answering these questions may help to personalise learning settings to the specific needs of the individual, to make optimal use of the beneficial effects of emotions on memory, and to alleviate the cognitive impairments stress and strong emotional responses may cause.
